# Estimated Blood Loss in Surgery for Thoracolumbar Fracture With Diffuse Idiopathic Skeletal Hyperostosis Using Percutaneous Pedicle Screws Compared to Surgery for Femoral Trochanteric Fractures

**DOI:** 10.7759/cureus.77984

**Published:** 2025-01-25

**Authors:** Megumu Kawai, Takeshi Sasagawa

**Affiliations:** 1 Orthopedic Surgery, Toyama Prefectural Central Hospital, Toyama, JPN

**Keywords:** diffuse idiopathic skeletal hyperostosis, femoral trochanteric fracture, hidden blood loss, percutaneous pedicle screws, thoracolumbar fracture

## Abstract

Introduction: Long posterior spinal stabilization is often needed for thoracolumbar fractures with diffuse idiopathic skeletal hyperostosis (DISH). Recently, surgery using percutaneous pedicle screws (PPS) has become more frequent. However, hidden blood loss (HBL) cannot be ignored in surgeries using PPS. The aim of this study was to measure estimated blood loss (EBL) including HBL in surgeries using PPS for thoracolumbar fractures with DISH, by comparing with EBL including HBL in surgeries for femoral trochanteric fractures, a common trauma among elderly people.

Materials and methods: Twenty-two patients who underwent surgery using PPS for thoracolumbar fracture with DISH were included (group D). Sixty-six patients with trochanteric fractures of the femur were matched to group D for age, sex, anticoagulant use, height, and weight and used as controls (group F). We evaluated intraoperative blood loss (IBL), EBL on the first and seventh postoperative days, and HBL on the first postoperative day. EBL was calculated based on hemoglobin values. IBL was collected from surgical records. HBL was calculated as the difference between EBL on the first postoperative day and IBL. Each variable was compared between groups.

Results: EBL was not significantly different between the two groups on either postoperative day one or day seven. IBL was significantly greater in group D than in group F. HBL was significantly less in group D than in group F. In group D, HBL accounted for 71 (%) of EBL on day one, whereas in group F, it accounted for 92 (%).

Discussion: Despite that surgery using PPS for thoracolumbar fractures with DISH requires fusions covering several vertebral levels, EBL was the same as in surgery for trochanteric fractures of the femur, and therefore, acceptable for elderly patients.

Conclusions: EBL in surgery for thoracolumbar fracture with DISH using PPS is comparable to that for trochanteric fractures of the femur.

## Introduction

Thoracolumbar fractures with diffuse idiopathic skeletal hyperostosis (DISH) often do not achieve bony fusion using conservative treatment [1]. Therefore, posterior spinal stabilization may be needed in such cases. Longer fusions spanning three levels above and three levels below are recommended because of the long lever arm associated with bony ankylosis among patients with DISH [2]. These patients are mostly elderly males and often have diabetes or obesity as an underlying condition [3]. Worldwide, mortality rates from 8% to 32% have been reported in this population using conventional open surgery [2,4]. In Japan, a mortality rate of 12% has been reported [5]. Hence, the invasiveness of conventional open surgery is an issue for elderly patients with underlying diseases.

Recently, posterior spinal stabilization with percutaneous pedicle screws (PPS) has become more frequent because it is less invasive. Intraoperative blood loss (IBL) during surgery using PPS is reported as 134-167 mL, significantly less than the 500-1240 mL in conventional surgery [5,6]. However, not only IBL but also hidden blood loss (HBL) cannot be ignored in surgery using PPS [7]. HBL is invisible blood loss caused by hemolysis and extravasation of blood into tissues [8]. Therefore, studies of estimated blood loss (EBL) including HBL are necessary in order to assess blood loss in surgery using PPS. To the best of our knowledge, there are no reports on EBL including HBL in posterior spinal stabilization using PPS for thoracolumbar fractures with DISH. Therefore, it is not clear whether EBL in this procedure is acceptable for surgeries involving the elderly population. The aim of this study was to examine whether EBL in posterior spinal stabilization using PPS for thoracolumbar fractures with DISH is reasonable for the elderly, by comparing it with EBL in trochanteric fractures of the femur, a common surgery for the elderly.

## Materials and methods

The medical ethics committee of our hospital approved the present study. All patients provided informed consent to use all patient data. This investigation was a retrospective study using electronic medical records. Twenty-two patients who underwent posterior spinal stabilization using PPS for thoracolumbar fracture with DISH between January 2015 and January 2023 were included (group D). Exclusion criteria included patients who underwent conventional open surgery, those with other trauma requiring surgery, those with hemothorax requiring a chest drain, and the lack of a complete data set. Fractures at various vertebral levels are shown in (Table 1).

**Table 1:** Level of injury and number of patients.

Injured level	Number of patients, n
T6	1
T7	1
T8/9	1
T9	2
T10	1
T11	3
T12	5
T12/L1	1
L1	5
L2	1
L3	1

Spinal stabilization with PPS was performed between three levels above and three levels below the fracture in all patients but not at the fracture level. Three patients also underwent posterior decompression. In these cases, posterior decompression with a median incision was performed after spinal stabilization with PPS.

A total of 66 patients with trochanteric fractures of the femur were matched to group D for age, sex, anticoagulant use, height, and weight and used as controls (group F). All 66 patients in group F underwent osteosynthesis. We evaluated IBL, EBL on the first and seventh postoperative days, and HBL on the first postoperative day. Each variable was compared between the study groups. IBL was evaluated by the measured suction loss plus the weight of loss in gauze. EBL was calculated using the Nadler formula [9] shown below based on Hb values. HBL was calculated as the difference between EBL on the first postoperative day and IBL. The Nadler formula is defined by

Women’s blood volume (L)=height (m)^3^×0.356+weight (kg)×0.033+0.183;

Men’s blood volume (L)=height (m)^3^×0.367+weight (kg)×0.032+0.604;

Hb_loss _(g)=blood volume (L)×(Hb_pre_ (g/L)-Hb_post_ (g/L))+26.5 (g)×(transfusion unit);

EBL (mL)=(Hb_loss_ (g)/Hb_pre_ (g/L))×1000;

HBL (mL)=EBL_day 1_ (mL)-IBL (mL);

Hb_pre_: Hb before surgery; Hb_post_: Hb one or seven days after surgery; EBL_day 1_: EBL on day one.

All data are expressed as mean ± standard deviation. A Mann-Whitney U test and a chi-square test were used to compare data. Differences with p<0.05 were considered significant. All statistical analyses were conducted using IBM SPSS Statistics for Windows (version 22; IBM Corp, Armonk, NY).

## Results

The average age was 77 years in group D and 78 years in group F. In both groups, 82% were male, and 23% took anticoagulant or antiplatelet medication. There were no significant differences in body size (Table 2).

**Table 2:** Patient background. Data presented as the mean ± standard deviation unless otherwise shown.

	Group D	Group F	P
Number	22	66	
Age, years	77.0±10.0	78.4±12.8	0.55
Male, n (%)	18 (81.8)	54 (81.8)	1.00
Height (m)	1.60±0.09	1.61±0.09	0.89
Weight (kg)	60.6±13.4	57.7±8.1	0.43
Anti-coagulant medication, n	5	15	1.00

**Figure 1:**
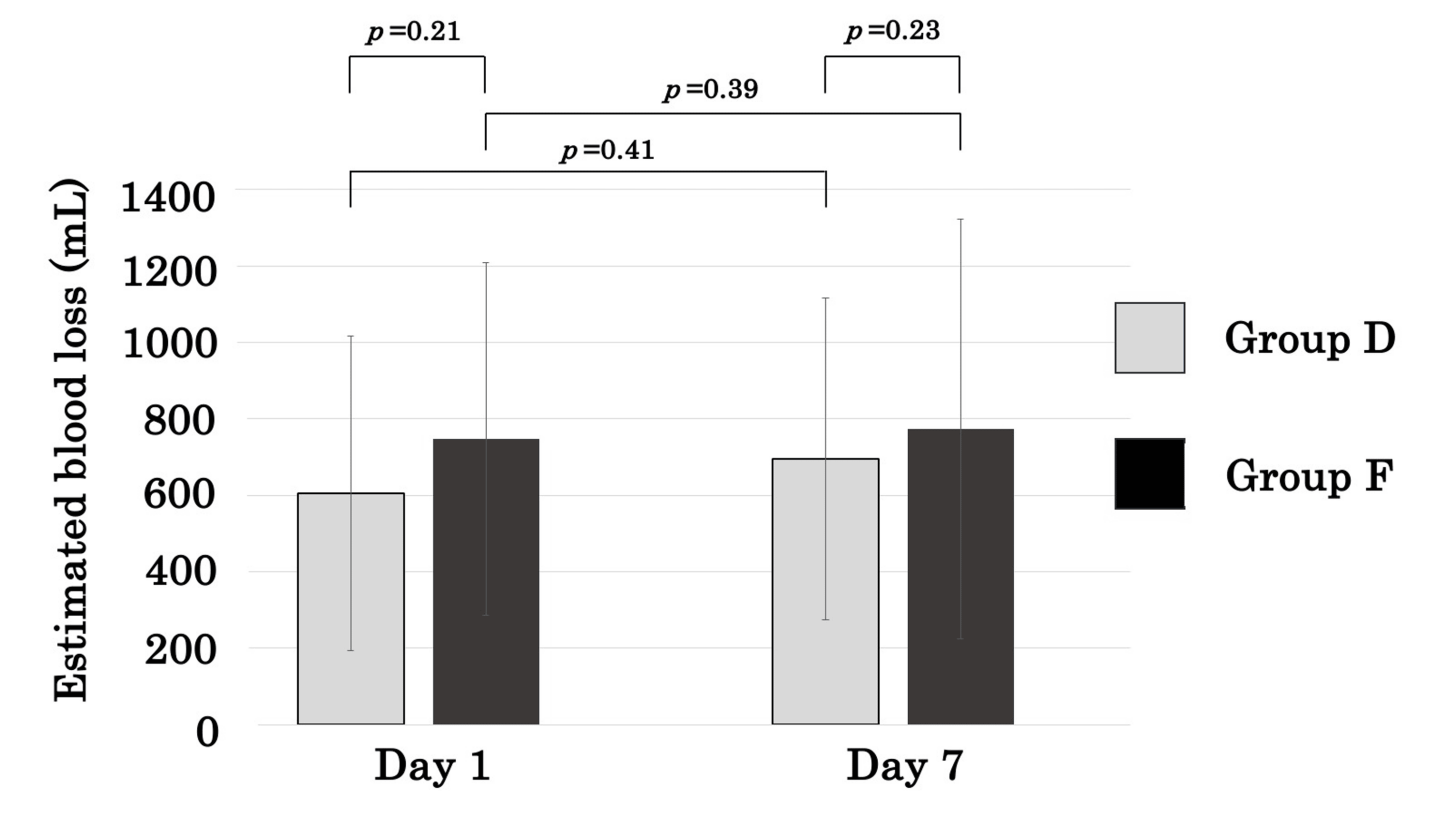
Comparisons of EBL between groups. EBL, estimated blood loss

**Figure 2:**
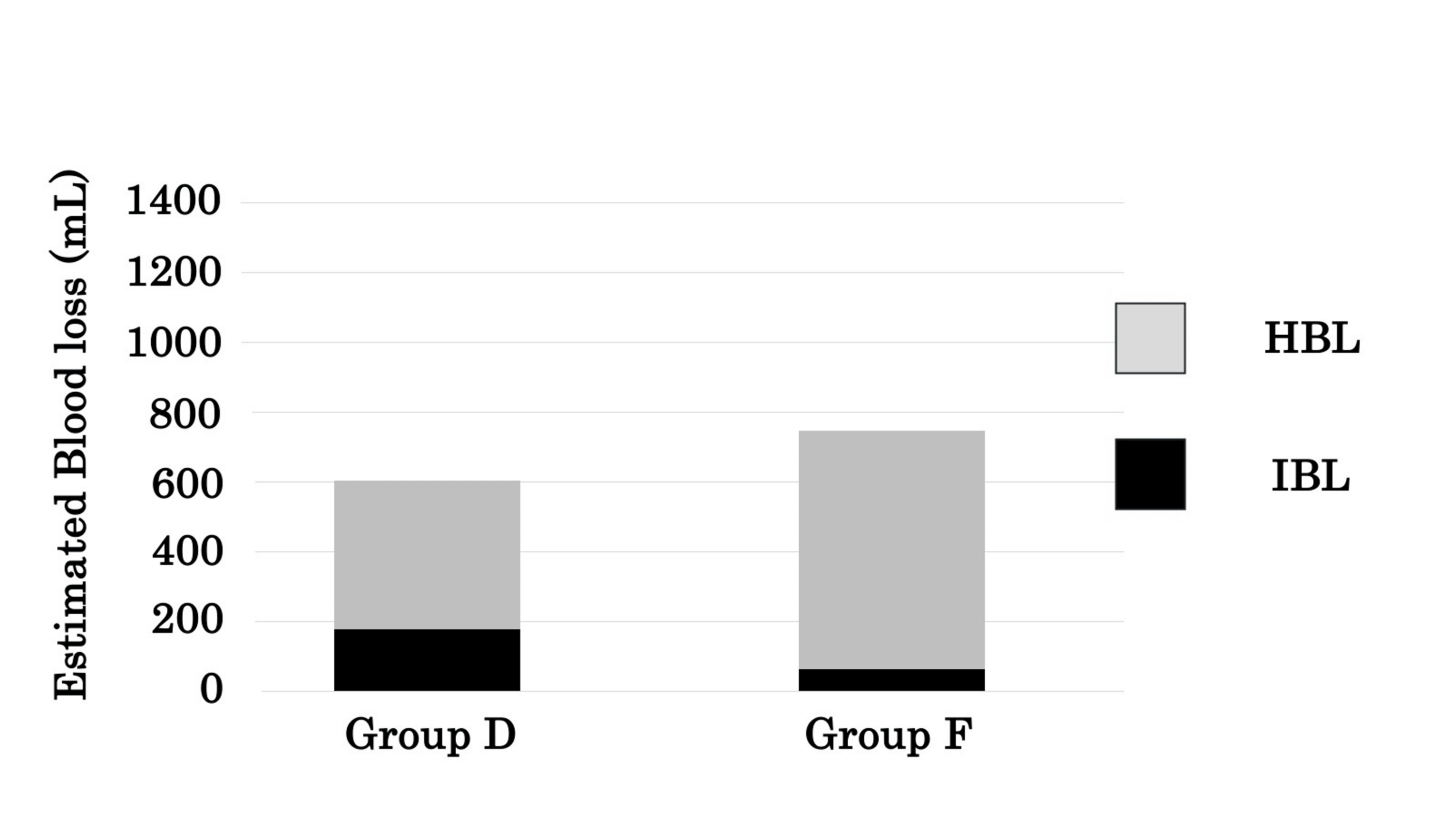
The percentage of EBL accounted for by HBL and IBL in groups D and F. EBL, estimated blood loss; IBL, intraoperative blood loss; HBL, hidden blood loss

EBL was not significantly different between the two groups on either postoperative day one or day seven (Figure 1, Table 3). Although EBL on day seven tended to be higher than on day one in both groups, the differences were not statistically significant (Figure 1). IBL in group D was significantly greater than in group F (p<0.01). In contrast, HBL in group D was significantly less than in group F (p=0.03, Table 3). In group D, HBL accounted for 71% of EBL on day one, whereas in group F, it accounted for 92% (Figure 2).

**Table 3:** Perioperative outcomes in each group. *p<0.05 EBL, estimated blood loss; IBL, intraoperative blood loss; HBL, hidden blood loss

	Group D (n=22)	Group F (n=66)	P
Hb before operation (g/dL)	12.3±1.6	11.6±2.0	0.88
Transfusion (units)	0.8±1.2	1.4±1.9	0.31
Hb on postoperative day 1 (g/dL)	10.4±1.6	9.6±1.8	0.03*
Hb on postoperative day 7 (g/dL)	10.8±1.4	9.8±1.5	0.01*
EBL on postoperative day 1 (mL)	605.1±410.4	747.7±461.5	0.21
EBL on postoperative day 7 (mL)	695.8±421.1	773.0 ±548.8	0.23
IBL (mL)	178.1±163.0	63.3 ±93.4	<0.01*
HBL (mL)	427.0±429.6	684.4 ±203.5	0.03*

## Discussion

EBL in posterior spinal stabilization using PPS for thoracolumbar fractures with DISH was not different from that in surgery for trochanteric fractures of the femur. Surgical treatment of thoracolumbar fractures with DISH often is performed due to the risk of nonunion and delayed paralysis associated with conservative management. Despite the fact that surgery using PPS for thoracolumbar fractures with DISH requires fusions covering several vertebral levels, EBL was the same as in surgery for trochanteric fractures of the femur, and therefore, acceptable for elderly patients.

Sheat et al. first reported the importance of HBL in orthopedic surgery for knee joint prostheses [10]. Recently, HBL in surgery using PPS for thoracolumbar fractures without DISH has also been reported several times [7,8,11]. In these studies, HBL accounted for 68-85% of EBL [7,8,11], which is very similar to the current study (71%).

In this study, EBL was not significantly different between groups. However, HBL in group F was greater than in group D, while IBL in group D was greater than in group F. Therefore, the etiologies of perioperative bleeding were different between the groups. HBL in surgery for spine fractures is correlated with the degree of fracture dislocation and reposition [12] and is caused by blood infiltration into the soft tissue or the postoperative muscle space [12]. Moreover, according to Zhou et al., muscle thickness is a risk factor for HBL [13]. In that study, most cases exhibited minimal preoperative dislocation. Fractures were mostly located at the thoracolumbar junction where the soft tissue is relatively thin. Therefore, despite a large IBL due to the length of the stabilization (three levels above and three levels below), HBL was not greater than in surgery for femoral trochanteric fractures. Ren et al. reported that topical use of tranexamic acid can reduce HBL in posterior lumbar spinal fusion surgery without significant complications [14]. However, there is no report of the effect of tranexamic acid for posterior stabilization using PPS for thoracolumbar fractures with DISH. Therefore, it is necessary to examine whether tranexamic acid is effective in reducing HBL in cases of DISH.

The importance of HBL in surgery for trochanteric fractures of the femur also has been reported [15-18]. Smith et al. reported that most of the HBL in proximal femoral fractures was preoperative [17]. Similarly, Guo et al. reported that intramedullary fixation does not cause a large amount of HBL in femoral fractures, rather bleeding from the fracture site is the main cause of HBL [18]. In this study, the proportion of HBL in EBL was 92% and the proportion of IBL was 8%, suggesting that bleeding was caused primarily by the fracture, not the surgery.

Transfusions of 0.8-1.4 units on average were required in both groups in the perioperative period. Sasagawa et al. reported that EBL on day seven tended to be less than on day one postoperatively when using PPS for traumatic thoracolumbar fractures in a younger population, and no patients required transfusion [11]. On the other hand, in our study, the average age was 77-78 years, relatively older than in their report. EBL on day seven tended to be higher than on day one in both groups. Zhou et al. reported that the amount of HBL is correlated with age [13], reasoning that bleeding is liable to infiltrate more easily into interstitial spaces and agglutinate there owing to muscle wastage [13,19]. Additionally, hematopoiesis in the elderly is lower than in the younger population [20]. Consequently, EBL may increase over time in the elderly because of the persistence of postoperative invisible bleeding and low hematopoiesis. Postoperative anemia in the elderly may lead to disorders of the brain, heart and kidneys, infections [21], and delirium [22]. Therefore, sustained monitoring of postoperative hemoglobin levels is essential in the elderly population.

There were some limitations to the present study. First, the sample size is small. If the sample size were larger, factors resulting in increased EBL might be examined. Second, the days between injury and surgery varied among patients so the assessment of surgical invasiveness is not only based on the amount of blood loss. To understand the blood loss in surgery using PPS for thoracolumbar fractures with DISH, further study is required.

## Conclusions

We measured EBL in posterior spinal stabilization for thoracolumbar fractures with DISH in elderly patients by comparing it with EBL in surgery for trochanteric fractures of the femur, a common trauma among elderly people. EBL in surgery for thoracolumbar fracture with DISH using PPS is comparable to that for trochanteric fractures of the femur. Therefore, even though surgery using PPS for thoracolumbar fractures with DISH requires fusions covering several vertebral levels, this is an acceptable procedure for elderly patients.
